# Learning *by* Heart or *with* Heart: Brain Asymmetry Reflects Pedagogical Practices

**DOI:** 10.3390/brainsci13091270

**Published:** 2023-08-31

**Authors:** Martin Schetter, David Romascano, Mathilde Gaujard, Christian Rummel, Solange Denervaud

**Affiliations:** 1 Department of Diagnostic and Interventional Radiology, Lausanne University Hospital, University of Lausanne, 1005 Lausanne, Switzerland; 2 Support Center for Advanced Neuroimaging (SCAN), University Institute of Diagnostic and Interventional Neuroradiology, Inselspital—Bern University Hospital, University of Bern, 3010 Bern, Switzerland

**Keywords:** brain asymmetry, cortical thickness, education, semantic memory, pedagogy, Montessori education

## Abstract

Brain hemispheres develop rather symmetrically, except in the case of pathology or intense training. As school experience is a form of training, the current study tested the influence of pedagogy on morphological development through the cortical thickness (CTh) asymmetry index (AI). First, we compared the CTh AI of 111 students aged 4 to 18 with 77 adults aged > 20. Second, we investigated the CTh AI of the students as a function of schooling background (Montessori or traditional). At the whole-brain level, CTh AI was not different between the adult and student groups, even when controlling for age. However, pedagogical experience was found to impact CTh AI in the temporal lobe, within the parahippocampal (PHC) region. The PHC region has a functional lateralization, with the right PHC region having a stronger involvement in spatiotemporal context encoding, while the left PHC region is involved in semantic encoding. We observed CTh asymmetry toward the left PHC region for participants enrolled in Montessori schools and toward the right for participants enrolled in traditional schools. As these participants were matched on age, intelligence, home-life and socioeconomic conditions, we interpret this effect found in memory-related brain regions to reflect differences in learning strategies. Pedagogy modulates how new concepts are encoded, with possible long-term effects on knowledge transfer.

## 1. Introduction

Are you more curious to know or to understand concepts? Your orientation toward learning may well reflect your schooling experience. There is a growing interest in the impact of pedagogy on child development [1], suggesting that how children learn *how to learn* shapes core mechanisms of learning. A way to deepen our understanding of the impact of schooling experience on brain development is to contrast groups of children from pedagogies with fundamental differences in their settings (e.g., traditional and Montessori). This bottom-up perspective may provide new insights about learning and development.

In traditional Swiss schools, students learn through adult-led lecture-type interactions. Feedback on their work is in the form of grades and quantitative assessments. Furthermore, students’ work time is divided into one-hour classes, interrupted by breaks. From a social point of view, students usually interact with their same-age peers during recess and little during work (i.e., www.plandetudes.ch, accessed on 29 August 2022). In contrast, in Montessori schools, students learn through self-directed activities. Feedback on their work is provided using the didactic Montessori materials, enabling students to self-correct without formal assessment. Furthermore, students’ work time is not interrupted for a minimum of 3 h. From a social point of view, students usually interact freely with peers of different age levels during work (i.e., https://montessori-ami.org, accessed on 29 August 2022). Learning strategies are inherently different at the cognitive and social levels (i.e., solicited memorizing skills).

When contrasting students experiencing traditional versus Montessori pedagogy, differences emerge at the behavioral level. For example, students experiencing Montessori pedagogy show higher cognitive outcomes, such as reading skills [2,3], executive abilities [4,5] and creative thinking skills [6,7,8], but also higher abilities to recognize and manage emotions [9,10,11], and more globally, academic outcomes [4,12].

Underlying neural processes are also impacted. A study on how students address errors and correct responses reveals differences in brain activation and neural connectivity [13]. In addition to global higher neural activity in brain regions implied in self-engagement, Montessori-schooled students tend to have greater functional connectivity between brain regions involved in error management and problem solving after incorrect trials. Conversely, traditionally schooled students tend to have greater functional connectivity between the brain regions related to memory (i.e., right hippocampus) and executive skills (i.e., prefrontal cortex) after correct responses. This suggests that core learning strategies mirror pedagogical practices: error management versus learning by heart. Finally, a recent study shows how brain dynamics are also modulated by pedagogy, especially within brain networks related to creative cognition. Traditionally schooled students exhibit higher intrafunctional connectivity in the region of the brain responsible for coordinating other brain networks (i.e., the salience network) than Montessori-schooled students. They seem to have a dynamic functional imbalance, spending more time in an introspective state (i.e., overengagement of the default mode network) rather than in an executive mode. Both these static and dynamic brain functional connectivities suggest a less flexible mode of thinking [14]. While these functional MRI-based studies revealed differences in students’ neural activities in given conditions, they are less informative about long-term neural reorganization. For the latter, voxel-based morphometry is a preferred approach [15].

Interindividual differences related to schooling experience could also be investigated using markers of hemispheric asymmetries. Studies on cortical thickness (CTh) reveal that both left and right hemispheres are remarkably symmetric [16,17], and even when comparing CTh asymmetry index (AI) between babies and adults, no differences are observed [18]. Consequently, CTh symmetry is a marker of a healthy brain [16,17], while CTh asymmetries are mostly observed in cases of abnormal brain development [19], learning disabilities [20], or illness [21]. However, experience also influences cortical thickness (CTh) [22]. In fact, experience-dependent plasticity leads to the emergence of subtle asymmetries within specific brain regions. One example is meditation; the prefrontal cortex and right anterior insula are thicker in participants exercising meditation than in matched controls. As these brain structures are known to be involved in self-awareness, the observed CTh AI reflects an increased capacity for internal states’ attention in meditators [23]. As experience-dependent plasticity is even higher across the school years than in adults [24], differences in left and right cortical thickness in Montessori versus traditionally schooled students could be revealed within brain regions related to *how* learning processes are trained.

Learning processes imply the prefrontal region for executive- and attention-related aspects [25], while declarative memory (i.e., conscious memory of facts and events) is handled by the medial temporal lobe, which comprises a system of anatomically related structures, including the parahippocampal (PHC) region [26]. Both regions have lateralized functions, and therefore, CTh asymmetries may appear in these brain regions. First, the left prefrontal cortex volume (i.e., dorsomedial cortex and orbitomedial cortex) is positively correlated with fluid intelligence scores [27]. The left dorsolateral prefrontal cortex and bilateral medial prefrontal cortex gray matter volumes correlate with attention-related impulsivity [28]. Using repetitive transcranial magnetic stimulation to test the contribution of the right and left dorsolateral prefrontal cortex, it was shown that stimulation of the right dorsolateral prefrontal cortex specifically results in transient dissociation, reducing spatial accuracy but increasing verbal accuracy [29]. Second, the PHC region is highly associated with memory formation, navigation and temporal dynamics [30]. There, contextual associations related to episodic memory activate the right PHC region [31,32], while associations with semantic knowledge activate the left PHC region [32,33,34]. Episodic memory relates to the ability to relive an event in the context in which it originally occurred, while semantic knowledge relates to facts about the world (i.e., generalization of concepts) [35]. Furthermore, language functions and word structure are correlated with the left lateralization of the PHC region [36], as well as visually encoded items, suggesting that memory recollection of the source elicits greater PHC activation than memory without recollection [37].

Based on past work revealing similar executive abilities among wealthy Swiss schoolchildren enrolled in either Montessori or traditional schools [4], we do not expect CTh AI differences within the prefrontal brain regions when comparing these groups of students. However, regarding the PHC region, we suspect that the opposed memorizing strategies found in Montessori versus traditional practices would reinforce the lateralization of one versus the other hemispheres. In fact, Montessori-schooled students excel in recognition tasks compared with traditionally schooled students [38]. Denervaud et al. (2019) further show that semantic networks are highly permeable to educational experience. They find that compared with students experiencing a traditional education, students experiencing Montessori education show a more flexible semantic network structure, characterized by higher connectivity and shorter paths between concepts, as well as lower modularity. Also, working memory is modulated in favor of Montessori-schooled students [2,3,4]. Finally, neural asymmetry has already been shown in functional connectivity in relation to pedagogy and memory: for correct answers, the traditional pedagogy group shows stronger connectivity between brain regions related to error monitoring (e.g., the anterior cingulate cortex and the right hippocampus [13]). Therefore, an asymmetry in cortical thickness of memory-related regions as a function of school experience may arise.

Here, we specifically explored differences in the CTh AI of the brains of healthy participants. For AI studies, CTh is a robust metric to use [17,39,40,41,42,43,44]. We first aimed at replicating and extending past work on CTh AI development by comparing students’ CTh AI with adults’ CTh AI at the whole-brain level. We hypothesized a null difference between the two groups. We further split the student participants’ dataset based on their schooling experience: the ones enrolled in Montessori versus the ones enrolled in traditional schools. Adopting a down-scaling approach, we explored CTh AI at the whole-brain, lobe-wise and subregions levels. Based on previous work contrasting students from Montessori and traditional schools revealing differences in memory-related skills and underlying neural connectivity [2,3,4,13], we expected asymmetry to be observed across development, specifically in the PHC subregion (to the left for Montessori pedagogy and to the right for traditional pedagogy) differences related to experience at school. These hypotheses are mainly based on the known functional lateralization of the PHC region [31,32,33].

## 2. Materials and Methods

### 2.1. Participants

In the framework of a large study on pedagogy and brain development that has been running since 2018, 3-to-18-year-old healthy participants are regularly recruited in local schools. Inclusion criteria comprise schooling background (i.e., traditional or Montessori pedagogy), age (3–18 y.o.), and no diagnosis of neurodevelopmental or learning disorders. At the time of this study, a total of 112 participants were recruited with neuroanatomical data: fifty-six experiencing Montessori pedagogy and fifty-six experiencing traditional pedagogy. One participant was excluded because of excessive movement (*n* = 1). The final sample size for the analyses consisted of 111 participants (mean age = 10.45, SD = 3.39, 62 girls, 9 left-handed, Table 1). Fifty-six participants were experiencing Montessori pedagogy (mean age = 10.02, SD = 3.13, 27 girls, 4 left-handed, Table 1) and 55 participants were experiencing traditional pedagogy (mean age = 10.91, SD = 3.61, 35 girls, 5 left-handed, Table 1). Oral consent from the participants and written approval from the their parents were collected. Participants were compensated with a voucher or a neuroscience book.

As part of the same study, adults are regularly recruited by word of mouth. At the time of this study, a total of 78 adults were recruited. Data from one participant was excluded because of incompatibility with the MR scanner, leading to a total of 77 adults (mean age = 29.44, SD = 10.28, 38 women). Because of bimodal age distribution, only 20-to-30-year-old adult images were retained for the current study. The final sample size consisted of 51 adults (mean age = 23.95, SD = 2.02, 28 women, Table 1). Written approval for each participant was collected.

The present study was conducted in accordance with the Declaration of Helsinki, and the local ethics committee (Commission d’Ethique Romande—Vaud) approved the study protocol (PB_2016-02008, 204/15).

### 2.2. Group Variables

In Switzerland, the free-access schooling system is based on a strictly applied traditional pedagogy, whereas schooling systems with Montessori pedagogy are not free of charge. This can potentially lead to a selection bias, as the participants who have access to the Montessori pedagogy may have a higher socioeconomic background. In order to counter-balance this selection bias, details about parents’ socioeconomic status, home environment and participants’ fluid intelligence were gathered.

The socioeconomic status (SES) [45] represents the level of wealth and the social standing of the participants’ family. This status was assessed by a questionnaire about the educational (ranging from 0 to 4) and professional (ranges from 0 to 4) levels of either both relatives or sole relative (in the case of lone-parent family situations). The answers were averaged to form the final SES score (Table 1).

The home environment variable (HEV) represents a participant’s home-life conditions. This score was assessed by extracting specific details from a tailor-made questionnaire filled out by the relatives. Information about green space accessibility, number of meals shared with the family, interest of the parent for pedagogy and extracurricular activities of the participant were included as a compound normalized score. Each piece of information contributed equally to the final score, represented by a percentage. For instance, the information about the number of meals shared with the family was assessed per week, on a number from 0 to 7. It was then turned into a percentage, taking one quarter of the final compound score, e.g., a child whose parents have a high interest in pedagogy, who shares six meals a week with their parents and has access to a garden and practices extracurricular activity will have an HEV score of 91.43% (Table 1).

The fluid intelligence variable (FIV) represents the capacity of the participant to analyze, think logically and solve problems in new circumstances. This measure was assessed by the paper-based black-and-white version of the Raven’s Progressive Matrices test (PM-47) [46]. The 15 min test is composed of 3 sets of 12 matrices, each of these matrices missing a part, and the participant was asked to complete each matrix with 1 of the 6 or 8 choices proposed. Summing all the correct answers built the fluid intelligence score, e.g., a child who has 23 correct answers out of the 36 trials will have an FIV score of 23 (Table 1). Furthermore, given the correlation between these variables and brain morphometry measures [47,48], sex and handedness ratios were statistically tested.

### 2.3. MRI Acquisition

The brain images were acquired at the Center for BioMedical Imaging (CIBM) of the Lausanne University Hospital (CHUV-UNIL) in Switzerland on a Siemens 3T Prisma-Fit MRI scanner with a 64-channel head coil. For each participant, a three-dimensional high-resolution isotropic image with the T1-weighted pulse method and the Magnetization-Prepared Rapid Acquisition Gradient sequence (MPRAGE) was acquired (TR = 2000 ms; TE = 2.47 ms; 208 slices; voxel size = 1 mm × 1 mm × 1 mm; flip angle = 8°; field of view = 256 mm × 256 mm).

### 2.4. MRI Preprocessing

Raw images were preprocessed through the neuroimaging freeware Freesurfer 6.0.1 (www.surfer.nmr.mgh.harvard.edu, accessed on 2 July 2022). The processing stream for structural data includes skull stripping, B1 bias field correction and gray–white matter segmentation in the first hand, followed by the reconstruction of cortical surface models thanks to the boundaries highlighted by the segmentation (gray–white boundary surface and pial surface). Then, the processing stream continues with the labeling of regions on the cortical surface and subcortical brain structures, and the nonlinear registration of the cortical surface of an individual with stereotaxic atlas. The Freesurfer stream for structural MRI data ceases with a statistics analysis of group morphometry differences. Following this stream, the morphometric variables were able to be extracted, and values for the frontal, temporal, parietal, occipital and cingulate lobes of the cerebrum were mapped from the regional results provided by the Desikan–Killiany atlas [49]. Given its location, the insula region was studied as a separate lobe. For each participant, automatic segmentation was visually inspected and validated.

### 2.5. Cortical Thickness Computation

Cortical thickness (CTh) was computed as the distance between the inner surface and the outer surface of the cortex at each location. Each location is modelized as a vertex at the surface [50]. The inner surface of the cortex is defined as the boundary between gray matter and white matter, and the outer surface is defined by the boundary between gray matter and pial matter [51]. Also assimilated as the pial surface, cortical surface area (CSA) is computed as the surface of pial matter encompassing the cortex. To correct for differences in surface area among the ROIs *R* of the Desikan–Killiany atlas, the weighted CTh (WCTh) of each lobe *L* was computed on both hemisphere as follows:

$$WCTh_{L}=\frac{\sum_{R\in L}CTh_{R}\;\cdot \;CSA_{R}}{\sum_{R\in L}CSA_{R}}$$

The same correction was applied to compute the WCTh at the whole-brain level on both hemispheres:

$$WCTh_{WB}=\frac{\sum_{R}CTh_{R}\;\cdot \;CSA_{R}}{\sum_{R}CSA_{R}}$$

### 2.6. Asymmetry Index Computation

The asymmetry index (AI) was computed at different anatomical levels, whole-brain, lobe-wise and subregions, adopting a down-scaling approach.

The index of asymmetry was computed as follows on the freeware RStudio (R Core Team, 2020).

With *rh* relating to the brain ROIs *R* of the Desikan–Killiany atlas on the right hemisphere and *lh* to its counterpart on the left hemisphere, the CTh AI of a region was computed as follows:

$$CThAI_{R}=\frac{WCTh_{R_{lh}}-WCTh_{R_{rh}}}{WCTh_{R_{lh}}+WCTh_{R_{rh}}}$$

The same computations was applied for the CTh AI at the lobe-wise and whole-brain level.

### 2.7. Statistical Analysis

#### Group and Demographic Variables

Between-group (Montessori and traditional) homogeneity was statistically tested. Multiple *t*-tests were performed on age, socioeconomic status, home environment and fluid intelligence variables. Binomial derivatives of the Pearson’s chi-squared tests were performed on categorical variables to verify whether the proportion of female and left-handed participants was comparable between the two groups. These tests were performed at a significance level α=0.05 and computed with the package *stats* of the freeware RStudio (R Core Team, 2020).

### 2.8. Asymmetry index

#### 2.8.1. Adult and Student Participant Comparison

CTh AI data for both the adults and all the students were controlled using a Shapiro–Wilk test ($p_{s}>0.87$). To test whether the CTh AI was similar across age groups, CTh AI metrics of the student participants were compared with the CTh AI metrics of the adult participants at the whole-brain level using a Student *t*-test. An analysis of covariance (ANCOVA) was also performed to confirm that age had no impact on the CTh AI between groups. These analyses were computed with the software Jamovi (Jamovi Project, 2018).

#### 2.8.2. Montessori- and Traditionally Schooled Participant Comparison

To investigate differences in CTh AI between Montessori- and traditionally schooled participants, multiple linear regression analyses were computed. Measures of CTh AI at three different levels (whole-brain, lobe-wise and subregions) were successively investigated with pedagogy, sex, SES, age and the interaction between age and pedagogy as factors. These analyses were computed with the software Jamovi (Jamovi Project, 2018).

## 3. Results

### 3.1. Group and Demographic Variables

No significant differences between pedagogy groups were found in the age, SES, fluid intelligence or home environment variables (all p>0.1, Table 2). The proportion of girls and left-handed participants did not significantly differ by pedagogy group (all p>0.1, Table 2).

### 3.2. Asymmetry Index

#### 3.2.1. Adult and Student Participant Comparison

At the whole-brain level, no significant difference in CTh AI means (t(160)=−1.08, p=0.28) was found between the adult ($\mu =2.51\times 10^{-3},\;\sigma =6.47\times 10^{-3}$) and the student group ($\mu =3.82\times 10^{-3},\;\sigma =4.02\times 10^{-3}$). Furthermore, the ANCOVA revealed no effect of age (F(1,158)=1.46,p=0.229), group (F(1,158)=2.17,p=0.142) or interaction between both (F(1,158)=1.76,p=0.186). Both approaches suggested a comparable CTh AI between adult and student participants.

#### 3.2.2. Montessori- and Traditionally Schooled Participant Comparison

1. Whole-brain analysis
  The multiple linear regression (MLR) model was computed to evaluate the CTh AI at the whole-brain level based on age, pedagogy, the interaction between age and pedagogy, SES and sex. A significant MLR model was found with an $R^{2}$ of 0.12 (p=0.027, Supplements: Table S1)
  The interaction between pedagogy (traditional and Montessori) and age significantly predicted CTh AI ($\beta =-9.23\times 10^{-4},\;p=0.048$, Supplements: Table S1, Figure S1). Considering the significant scattering of the values, the CTh AI showed rather constant positive value for Montessori-schooled participants, while the CTh AI of traditionally schooled participants developed from a positive value toward a null value. This CTh asymmetry was observed toward the left hemisphere for Montessori-schooled participants, while traditionally schooled participants developed from a thicker cortex on the left toward higher symmetry. Except for the intercept, no other factors were reliably related to CTh AI (all p>0.056, Supplements: Table S1). While no main effect of pedagogy was observed, its significant interaction with age suggested differences at the lobe level. We further explored CTh AI using a down-scaling approach for the four main lobes, investigating each lobe individually (i.e., frontal, parietal, temporal, and occipital).
2. Lobe-wise analysis
  The only MLR model computed at the lobe-wise level revealed to be significant was related to CTh asymmetries across development at the whole brain or the temporal lobe, with an $R^{2}$ of 0.13 (p=0.017, Table 3).

**Figure 1:**
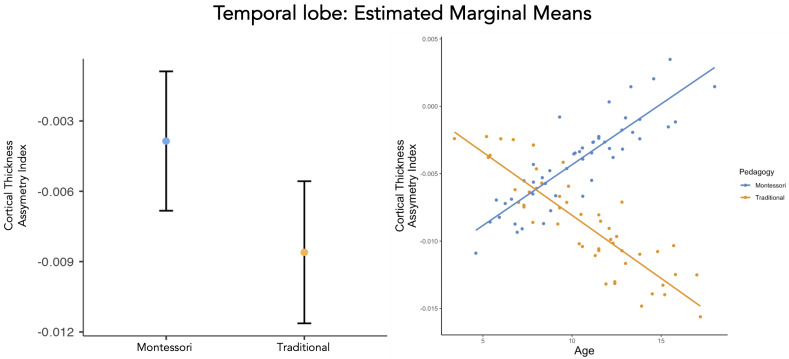
CTh AI of the temporal lobe (**left**) and its development (**right**) for Montessori− and traditionally schooled participants: Data points are the values predicted by the model that takes into account the effects of gender and SES.

There was a main effect of pedagogy (traditional and Montessori) on the CTh AI ($\beta =1.65\times 10^{-2},\;p=0.030$, Table 3, Figure 1). Overall, the CTh AI in the temporal lobe of Montessori-schooled participants ($\mu =-3.86\times 10^{-3},\;SE=1.53\times 10^{-3}$) was lower than the CTh AI in the temporal lobe of traditionally schooled participants ($\mu =-8.60\times 10^{-3},\;SE=1.50\times 10^{-3}$). However, when applying an FDR correction to compensate for multiple comparisons, this effect fades out ($p_{fdrcorrected}=0.12$), revealing that while a group difference is observed in the regression model, it is not strong. The interaction between pedagogy (traditional and Montessori) and age related to the CTh AI of the temporal lobe was significant and robust ($\beta =-2.04\times 10^{-3},\;p=0.004$, $p_{fdrcorrected}=0.016$, Table 3, Figure 1). At the lobe level, consistent with the whole-brain effect, we report a developmental difference. Across development, Montessori-schooled participants showed a CTh AI in the temporal lobe toward the left hemisphere, compared with the traditionally schooled participants who developed a CTh AI toward the right hemisphere in the temporal lobe. However, age alone did not predict CTh AI in the temporal lobe ($\beta =9.22\times 10^{-4},\;p=0.068$, Table 3) and neither did other factors (p>0.068, Table 3).

3. Subregions of the temporal lobe analysis
  Our hierarchical testing paradigm allowed us to further explore the nine subregions of the temporal lobe, adopting the same modeling approach. The only MLR model computed at the subregion level revealed to be significant was for the PHC subregion, with an $R^{2}$ of 0.11 (p=0.047, Table 4).

**Figure 2:**
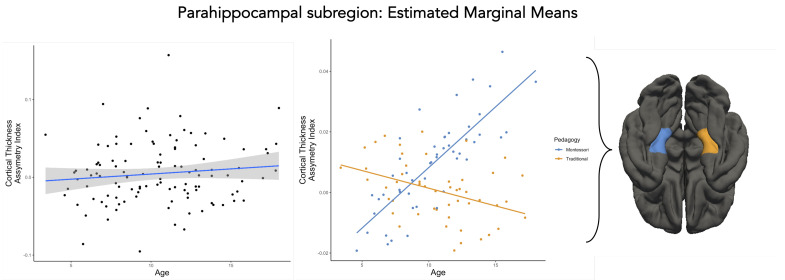
Development of the CTh AI of the PHC subregion for the whole sample (**left**) and for Montessori− and traditionally schooled participants (**right**): Data points are the values predicted by the model that takes into account the effects of gender and SES.

There was a main effect of age related to CTh AI in the PHC subregion ($\beta =4.16\times 10^{-3},\;$p=0.025, Table 4, Figure 2). Through development, the CTh AI increased toward the left hemisphere for the PHC subregion. However, this effect was not robust to correction for multiple comparison ($p_{fdrcorrected}=0.186$) Furthermore, there was an interaction between pedagogy (traditional and Montessori) and age ($\beta =-6.09\times 10^{-3},p=0.017$, Table 4, Figure 2). Montessori-schooled participants had a significant increase in CTh AI within the left PHC subregion compared with the traditionally schooled group, which showed an increase toward the right PHC subregion. This effect drops to the trend level when correcting for multiple comparisons ($p_{fdrcorrected}=0.066$). No other factors were reliably related to CTh AI in the PHC subregion (p>0.053, Table 4).

## 4. Discussion

Neuroplasticity, the capacity of the brain to organize neural activity through development and experience, is high across the school years [24]. This experience-dependent plasticity can be studied through different lenses, such as tractography [52], functional connectivity [53] or morphometry [23,54]. While environmental factors like family environment or intense training (e.g., music practice [55]) are known to modulate brain anatomy [56], little is known about schooling experience. More specifically, recent work has shown that while the cortical thickness (CTh) of the left and right hemispheres is globally similar (i.e., symmetric), between-hemisphere variations (i.e., asymmetry index) can be observed, and they provide insights about typical or atypical neurodevelopment [17,19,57,58]. The current study specifically contrasted CTh asymmetries in 4–18 y.o. participants enrolled in Montessori or traditional schools.

At the whole-brain level, the CTh AI was similar between the adult group and the student group, even when controlling for age. While hemispheres are globally symmetric across ages, the pedagogy experienced across development was related to CTh asymmetries at the whole-brain level. More precisely, this effect was driven by the temporal lobe within the PHC subregion. While Montessori-schooled participants showed an asymmetry toward the left hemisphere, traditionally schooled participants showed an asymmetry toward the right hemisphere. We interpret this effect in memory-related brain regions to reflect the differences in learning strategies found in Montessori versus traditional settings.

Past work on brain asymmetry was mostly performed using adult participants’ data, reporting rather symmetric hemispheres [16,17]. While other studies have investigated asymmetry in atypical children [19,57,58], few report developmental data on CTh asymmetry metrics. Hill et al. (2010) compared healthy babies with healthy adults and found no difference in terms of cortical folding patterns and hemispherical depth asymmetries. Consequently, we first aimed at extending past work. No difference in CTh asymmetry between the adult group and the student group was found on data acquired with the same MR scanner and the same acquisition sequence, in line with past work [59]. Hemispheres are similarly symmetric, suggesting that the left and right sides of the cortex develop globally concomitantly. We further use brain asymmetries as possible markers of inter-individual variabilities in healthy participants, related more to experience-dependent plasticity.

Here, depending on the pedagogy participant’s experienced, CTh asymmetry was observed at the whole-brain level across development. Scaling down the analyses, we found this effect to be driven by the PHC region within the temporal lobe. The PHC region is recruited for associative memory, binding contextual items with information (e.g., face–name) [30,60,61]. For participants enrolled in Montessori schools, we report a CTh asymmetry toward the left hemisphere and the reverse pattern for participants enrolled in traditional schools (i.e., toward the right hemisphere). Past work reports the right PHC region to be activated for associations with episodic memory and spatial memory [31,32], while the left PHC region is more active in associations with new semantic information and verbal memory [32,33,34]. Our results suggest that participants experiencing traditional pedagogy develop more contextual associations related to episodic memory, while participants experiencing Montessori pedagogy favor contextual associations related to semantic memory. This seems consistent with the environment in which children learn. Indeed, in traditional pedagogy, students learn concepts in a disjointed way (e.g., a concept of geometry and later a concept of geography are distinctly introduced). It is therefore harder to associate different concepts, and learning becomes a succession of unrelated information to remember. This is in line with the neural connectivity toward the right hippocampus observed during an MRI math task in traditionally versus Montessori-schooled students [13]. Each piece of learning is then associated not with another piece of knowledge but with the spatiotemporal context in which the student was. In this way, the learned information is associated with a fixed context, which strongly limits knowledge transfer or generalization. Furthermore, if the school context is perceived as stressful because of grades, exams and competitive settings, it influences learning and memory as well [62]. While no differences in prefrontal cortical thickness was observed [63], it is possible that stress induces more fixed than flexible memory [64]. Future studies should investigate the relation between stress- and memory-related brain structures. Given these interesting results, future work should also unveil the effect of duration of exposure or narrow age range to better understand how knowledge is built across time and experience.

Conversely, in Montessori pedagogy, the manipulation of sensory didactic materials was designed to allow students to sense abstract notions, facilitating access to the meaning of information [13]. Furthermore, mixed-age classes encourage peer-to-peer learning; students must rephrase what they have learned and understood, forcing them to understand concepts and thus higher-level associations. Finally, each student has the freedom to choose their activity, which is usually set in an interdisciplinary manner (e.g., a concept of geometry is related to a concept of geography; Pythagoras and Greece). In this way, knowledge is encoded and embedded to be transferable to other and more abstract concepts. New knowledge is introduced only when the student is ready to get it, which differs from one student to another. Associations may be more easily made with the learned semantic information, reflected in CTh asymmetry toward the left hemisphere. This view corroborates previous behavioral studies on semantic memory showing that students experiencing Montessori pedagogy present a more flexible network structure, characterized by higher connectivity and shorter paths between concepts [6], and higher working memory scores [2,4] than students experiencing traditional pedagogy [6]. Together, it seems that school pedagogy modulates memory at the behavioral, semantic network and anatomical levels.

This study has a few limitations that need to be stated. First, our study had a cross-sectional design, refraining from any causal developmental claims. Future work should target a longitudinal study to confirm and deepen our work. It could also be of interest to investigate CTh AI in adults, taking into account their schooling experience, to evaluate whether asymmetries are still observed or not. Second, the sample size is relatively low for the large number of statistical tests computed, and future work should increment it. However, we were able to review images manually to ensure high-quality data only, with a uniform acquisition protocol in the 188 participants. Third, while our study investigated differences in Montessori- versus traditionally schooled participants, a selection bias could not be avoided. To keep it as low as possible, we gathered information about participants’ family backgrounds using a long and detailed questionnaire. Nevertheless, similar work should be replicated in a country where Montessori schools exist in public settings to get rid of any parent-related bias. Finally, the quality of adjustment of the MLR models was, respectively, 0.12, 0.13 and 0.11, meaning that while pedagogy partially explains CTh AI within the PHC region, most of the factors impacting it are still to be discovered. For example, hormonal factors may have an important influence on this variability as well [65].

## 5. Conclusions

We investigated the differences between students enrolled in Montessori versus traditional schools in terms of CTh asymmetry. The main differences were found within the PHC subregions of the temporal lobe, highlighting a rightward CTh asymmetry in participants experiencing traditional pedagogy and a leftward asymmetry in participants experiencing Montessori pedagogy. The PHC region, previously claimed to be associated with memory and visuospatial processing, is functionally lateralized: the left PHC region is associated with semantic context encoding, while the right PHC region is associated with episodic context encoding. This suggests that students who learn with Montessori pedagogy have a higher tendency to make connections between concepts, fostering deep learning and knowledge transfer. Conversely, students who learn with traditional pedagogy tend to memorize knowledge in a fixed way, restricted to a given situation. These orientations may have long-term effects on flexible knowledge transfer and critical or creative thinking. In a society where artificial intelligence increments daily, the experience of concepts in an interdisciplinary way seems an asset. We are in an era where learning *with* heart is more promising than learning *by* heart. These results have direct implications for practitioners.

## Figures and Tables

**Table 1:** Age and group variables for adults, Montessori- and traditionally-schooled participants: SES stands for socioeconomic status, FIV stands for fluid intelligence variable and HEV stands for home environment variable.

Group				
**Variable**	**Age**	**SES**	**FIV**	**HEV**
**Adults**				
Mean	23.95	-	-	-
SD	2.02	-	-	-
Min	20.83	-	-	-
Max	29.58	-	-	-
Missing	-	-	-	-
**Montessori-schooled**				
Mean	10.0	3.1	32.9	93.5
SD	3.1	0.5	4.0	10.2
Min	4.6	1.8	20.0	66.7
Max	18.0	4.0	36.0	100.0
Missing	-	5	4	6
**Traditionally schooled**				
Mean	10.91	3.05	32.83	91.89
SD	3.61	0.64	3.57	11.20
Min	3.4	1.75	19	58.33
Max	17.83	4	36	100
Missing	-	5	7	7

**Table 2:** Statistic tests of demographic and group variables for Montessori- and traditionally schooled participants testing the difference in samples; statistics used were all *t*-tests, except for sex and handedness, where Pearson’s chi-squared tests were used.

*Statistic Tests*				
**Variable**	***Test Statistic***	***Degree of Freedom***	***p-Value***	***Effect Size***
Sex	2.68	1	0.10	-
Handedness	0.141	1	0.71	-
Age	−1.39	109	0.17	−0.26
Socioeconomic Status	0.40	99	0.70	0.08
Fluid Intelligence	0.95	98	0.95	0.01
Home Environment	0.45	96	0.45	0.15

**Table 3:** Coefficients of the MLR model related to the CTh AI of the temporal lobe: M stands for Montessori; T stands for Traditional; Age × Pedagogy stands for the interaction term. Significant terms are highlighted in bold.

*Statistics of the Model*				
**Variable**	***F***	$R^{2}$	***Degrees of Freedom***	***p-Value***
CTh AI	2.92	0.13	5, 95	0.017
***Coefficients of the Model***				
Variable	*β-coefficient*	*SE*	*Test statistic*	*p-value*
Intercept	−4.33 × 10^−3^	7.84 × 10^−3^	−0.55	0.582
Age	9.22 × 10^−4^	5.00 × 10^−4^	1.85	0.068
**Pedagogy (T-M)**	1.65 × 10^−2^	7.5 × 10^−3^	2.20	0.030
Sex (♂-♀)	−5.91 × 10^−4^	2.16 × 10^−3^	−0.27	0.785
SES	−2.88 × 10^−3^	1.89 × 10^−3^	−1.53	0.130
**Age × Pedagogy (T-M)**	−2.04 × 10^−3^	6.89 × 10^−4^	−2.95	0.004

**Table 4:** Coefficients of the MLR model related to the CTh AI of the PHC subregion: M stands for Montessori; T stands for Traditional; Age × Pedagogy stands for the interaction term. Significant terms are highlighted in bold.

*Statistics of the Model*				
**Variable**	***F***	$R^{2}$	***Degrees of Freedom***	***p-Value***
CTh AI	2.34	0.11	5, 95	0.047
***Coefficients of the Model***				
Variable	*β-coefficient*	*SE*	*Test Statistic*	*p-value*
Intercept	1.23 × 10^−2^	2.86 × 10^−2^	0.43	0.667
**Age**	4.16 × 10^−3^	1.83 × 10^−3^	2.28	0.025
Pedagogy (T-M)	5.31 × 10^−2^	2.74 × 10^−3^	1.94	0.055
Sex (♂-♀)	−7.55 × 10^−3^	7.9 × 10^−3^	−0.95	0.342
SES	−1.35 × 10^−2^	6.89 × 10^−3^	−1.96	0.053
**Age × Pedagogy (T-M)**	−6.09 × 10^−3^	2.52 × 10^−3^	−2.42	0.017

## Data Availability

Data will be shared by the authors upon request to the corresponding author.

## Supplementary Materials

The following supporting information can be downloaded at: https://www.mdpi.com/article/10.3390/brainsci13091270/s1, Figure S1: Development of the CTh AI in the whole brain for Montessori- and tradiGonally schooled parGcipants: Data points are the values predicted by the model that take into account the effects of gender and SES; Table S1: Coefficients of the MLR model related to the CTh AI at the whole-brain level: M stands for Montessori; T stands for TradiGonal; Age X Pedagogy stands for the interacGon term. Significant terms are highlighted in bold.
