# Supplementary material for: Learning by Heart or with Heart: Brain Asymmetry Reflects Pedagogical Practices
Source: Brain Sci. 2023 Aug 31;13(9):1270. doi: 10.3390/brainsci13091270 (PMC10526483; doi:10.3390/brainsci13091270)
Supplement: Supplementary file 1 [file brainsci-13-01270-s001.zip › brainsci-2553178-supplementary.pdf]

SUPPLEMENTARY INFORMATION

Learning *By* Heart Or *With* Heart: Brain Asymmetry Reflects Pedagogical Practices

Martin Schetter, David Romascano, Mathilde Gaujard, Christian Rummel & Solange Denervaud

Statistics of the Model Variable	*F*	R^2	*Degrees of Freedom*	*p-value*
CThAI	2.67	0.12	5, 95	0.027

Coefficients of the Model Variable	*β-coefficient*	*SE*	*Test statistic*	*p-value*
**Intercept**	$\mathbf{1.22 \times 10^{-2}}$	$\mathbf{5.23 \times 10^{-3}}$	**2.35**	**0.021**
Age	8.25×10^{-5}	3.33×10^{-4}	0.25	0.805
Pedagogy (T-M)	8.00×10^{-3}	5.00×10^{-3}	1.60	0.113
Sex (♂-♀)	-2.27×10^{-3}	1.44×10^{-3}	-1.57	0.119
SES	-2.43×10^{-3}	1.26×10^{-3}	-1.93	0.056
**Age X Pedagogy (T-M)**	$\mathbf{-9.23 \times 10^{-4}}$	$\mathbf{4.60 \times 10^{-4}}$	**−2.01**	**0.048**

Table S1 Coefficients of the MLR model related to the CTh AI at the whole-brain level: M stands for Montessori; T stands for Traditional; Age X Pedagogy stands for the interaction term. Significant terms are highlighted in bold.

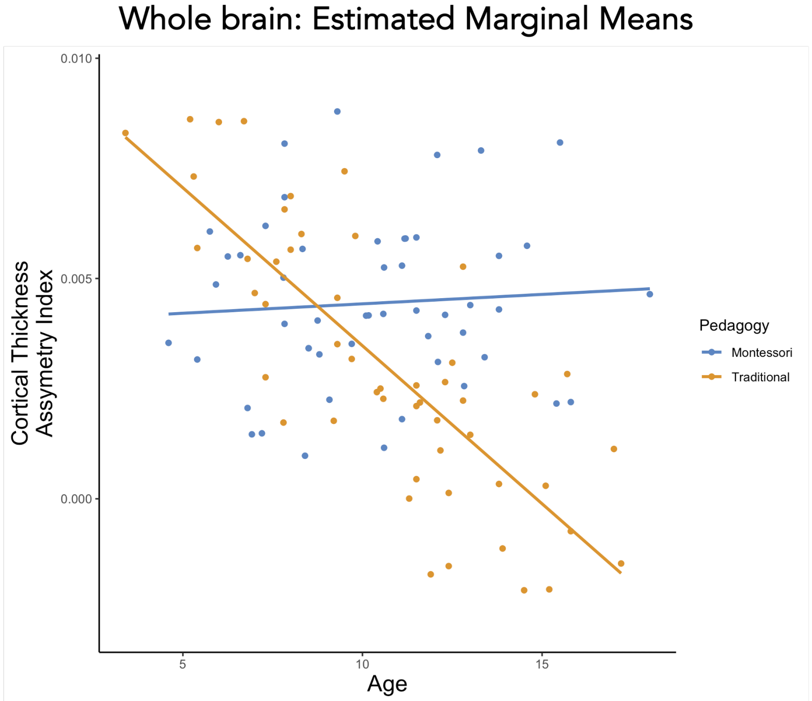

Figure S1 Development of the CTh AI in the whole brain for Montessori- and traditionally schooled participants: Data points are the values predicted by the model that take into account the effects of gender and SES.
